# 

**Published:** 1863-02

**Authors:** 


THE CHICAGO

### MEDICAL JOURNAL.

DANIEL BRAINARD, M. D.,
J. ADAMS ALLEN, M. D.,
Editors and Proprietors.

FEBRUARY, 1863.

CHICAGO:
J. C. W. BAILEY, BOOK AND JOB PRINTER, 180 CLARK STREET.
1863.

